# Association Between Subconjunctival Hemorrhage and Acute Coronary Syndrome: A 14-Year Nationwide Population-Based Cohort Study

**DOI:** 10.3389/fcvm.2021.728570

**Published:** 2021-10-01

**Authors:** Ping-Hao Chiang, Jung-Nien Lai, Yun-Chi Chiang, Kai-Chieh Hu, Min-Yen Hsu, James Cheng-Chung Wei

**Affiliations:** ^1^Institute of Medicine, Chung Shan Medical University, Taichung, Taiwan; ^2^Department of Allergy, Immunology & Rheumatology, Chung Shan Medical University Hospital, Taichung, Taiwan; ^3^School of Chinese Medicine, College of Chinese Medicine, China Medical University, Taichung, Taiwan; ^4^Department of Chinese Medicine, China Medical University Hospital, Taichung, Taiwan; ^5^Management Office for Health Data, China Medical University Hospital, Taichung, Taiwan; ^6^College of Medicine, China Medical University, Taichung, Taiwan; ^7^Department of Ophthalmology, Chung Shan Medical University Hospital, Taichung, Taiwan; ^8^Biotechnology Center, National Chung Hsing University, Taichung, Taiwan; ^9^Graduate Institute of Integrated Medicine, China Medical University, Taichung, Taiwan

**Keywords:** subconjunctival hemorrhage, acute coronary syndrome, population-based cohort study, Taiwan National Health Insurance Research Database, aspirin

## Abstract

**Purpose:** Subconjunctival hemorrhage (SCH) is usually a benign ocular disorder that causes painless, redness under the conjunctiva. However, since SCH and acute coronary syndrome (ACS) share many vascular risk factors, studies have suggested that these two disorders may be significantly associated with each other, and evaluate the concomitance of ACS in patients with SCH.

**Methods:** This population-based cohort study, enrolled 35,260 Taiwanese patients, and used the Taiwan National Health Insurance Research Database to identify patients with ACS and SCH. Outcomes were compared between the with and without SCH groups. The study population was followed until the date of ACS onset, the date of withdrawal, death, or December 31st 2013, whichever came first.

**Results:** Of the 85,925 patients identified with SCH between 1996 and 2013, 68,295 were excluded based on the study's exclusion criteria, and a total of 17,630 patients with SCH who were diagnosed by ophthalmologists between 2000 and 2012 were eligible for analysis. After 1:1 propensity score matching for 5-year age groups, gender, and the index year, the results showed that SCH was more common in the 40–59 age group (53.82%) and females (58.66%). As for the ACS-related risk factors, patients with diabetes mellitus (aHR = 1.58, 95% CI = [1.38, 1.81]), hypertension (aHR = 1.71, 95% CI = [1.49, 1.96]) and patients taking aspirin (aHR = 1.67, 95% CI = [1.47, 1.90]) had a notably higher risk of ACS. However, it was found that there were no significant differences in the occurrence of ACS between the non-SCH and SCH patients.

**Conclusion:** This results of this study regarding the risk factors and epidemiology of SCH and ACS were in keeping with previously reported findings. However, the results revealed no significant association between SCH and ACS.

## Introduction

Subconjunctival hemorrhage (SCH) is a common occurrence in emergency departments and outpatient clinics. Under most circumstances, SCH is a self-limiting disease that typically causes acute but painless ocular redness; a reduction in visual acuity is a rare occurrence. However, the occurrence of a red eye can trigger the patient's self-awareness of their eye and cardiovascular health, and can lead them to seek medical help, so ophthalmologists and physicians are often faced with SCH patients during their lifetime clinic practice (1).

A previous study conducted over 2 years by Cagini et al. (2) evaluated the causes of patient visits to the Eye Emergency Department. Of the 10,090 patients included in the study, most had ocular infection or trauma; 11.7% had SCH, which made SCH the third cause of patient visits. Furthermore, a study by Channa et al. (3) categorized the visits of 11,929,955 eye-related emergency department patients from 2006 to 2011, and SCH was responsible for just 3% of ocular problems across the United States.

SCH has some typical clinical features, such as the acute but normally painless appearance of redness under the conjunctiva, however it can often be asymptomatic (4). Although SCH doesn't usually cause any pain, bleeding under the conjunctiva still terrifies most patients, due to the obvious redness in the eye, which can even impact their daily social activities. Furthermore, recurrent SCH can be an indicator of underlying risk factors for cardiovascular diseases, which seem to make this red-eye syndrome more hazardous to the patients. Typical causes of SCH include orbital injuries, acute conjunctiva inflammation, conjunctival tumors, ocular surgeries and contact lens usage (4). These risk factors for SCH can be further divided into traumatic and spontaneous causes, representing two different clinical pathways for physicians to diagnose and evaluate. Contact lens wearing is a common reason for traumatic SCH as when damaged they can cause trauma directly to the conjunctiva during wearing.

Acute coronary syndrome (ACS) is a major health issue in developed countries, as it requires a large amount of caregiving and can be a heavy economic burden for both families and the country (5). The mortality and morbidity of ACS are noteworthy. ACS is a significant cause of death and disability in the Asia-Pacific region (6) with an in-hospital mortality exceeding 5%. Cardiovascular diseases can also show significant clinical symptoms in the patients' eyes due to the similarity between cardiovascular risk factors in different organs, meaning that the two common diseases, SCH and ACS, may often be linked together by clinicians.

It has been shown that SCH has a significant association with both gastrointestinal bleeding and dermatologic vasculature diseases (DVDs) (7). A 57-year-old man exhibited recurrent SCH for ~1 year before he was diagnosed with adenocarcinoma, without any family history of the condition (8). Another retrospective cohort study using the Taiwan National Health Insurance Research Database (NHIRD) revealed that patients with DVDs may have a higher risk of SCH, as the control group developed SCH with a significantly higher adjusted hazard ratio (aHR) of 2.69 compared with the study group (*p* < 0.05). If spontaneous SCH recurs in the patient within 1 month, the physician should check their coagulopathy profiles to identify any bleeding irregularities. Considering that many common risk factors of SCH are related to vascular or cardiovascular disabilities, the authors wondered if there was any correlation between SCH and ACS.

## Materials and Methods

### Data Source

The datasets used in this study are held by the Taiwan Ministry of Health and Welfare (MOHW). The Ministry of Health and Welfare must approve our application to access the data. Any researcher interested in accessing these datasets can submit an application form to the Ministry of Health and Welfare requesting access. Please contact the staff of MOHW (Email: stcarolwu@mohw.gov.tw) for further assistance. Taiwan Ministry of Health and Welfare Address: No.488, Sec. 6, Zhongxiao E. Rd., Nangang Dist., Taipei City 115, Taiwan (R.O.C.). Phone: +886-2-8590-6848. The authors confirm that they had no special access privileges others would not have.

Claims data of 1 million beneficiaries used in this retrospective cohort study was randomly sampled form the Taiwan National Health Insurance Research Database (NHIRD) and was named as the Longitudinal Health Insurance Database (LHID). The NHIRD was derived from a single-payer National Health Insurance (NHI) program launched in Taiwan on Mar. 1st, 1995. So far, there were ~99.9% of residents in Taiwan in the healthcare system. It contains the information regarding gender, date of birth, enrollment and withdrawal dates, dates of inpatient and outpatient encounters, the International Classification of Diseases, 9th Revision, Clinical Modification (ICD-9CM) codes for diagnoses and procedures, and details of prescription drugs. De-identification and anonymization were used to protect the privacy of beneficiaries in the database. Therefore, patient consent was not required to access the NHIRD. This study was approved by the Research Ethics Committee at China Medical University and Hospital [CMUH104-REC2-115(AR-4)].

### Study Population

The aim of this retrospective cohort study is to explore the association of subconjunctival hemorrhage (SCH) (ICD-9-CM: 372.72) with acute coronary syndrome (ICD-9-CM: 410, 411, and 413). Only patients diagnosed with SCH by ophthalmologists and with at least one hospital admission or at least two outpatient encounters between 2000 and 2012 were included in this study, and they were defined as cases. Others were defined as controls. The study population was followed until the date of acute coronary syndrome onset, the date of withdrawal or death, or December 31st, 2013.

The index date for cases was the date of initial diagnosis of SCH, and the index date for controls was the random pseudo-diagnosis date between 1996 and 2013. The index date for the control group was randomly allotted the date between 2000 and 2013.The exclusion criteria for the case and control groups are as follows: (a) patients with traumatic SCH (ICD-9-CM: 850-854, 871, 921 and 959.01); (b) index date not between enrollment and withdrawal date; (c) patients with acute coronary syndrome before index date; (d) patients aged <20 years; (e) follow-up time less than or equal to 0; (f) missing values for sex.

Comorbidities involved are as follows: chronic obstructive pulmonary disease (COPD) (ICD-9-CM: 490 to 496), diabetes mellitus (ICD-9-CM: 250), hypertension (ICD-9-CM: 401 to 405), hyperlipidemia (ICD-9-CM: 272.0), chronic kidney disease (ICD-9-CM: 403, 404, 585, and 586), chronic liver disease (ICD-9-CM: 571.4) and hemorrhagic condition (ICD-9-CM: 287). Medication considered is aspirin. Patients with at least one hospital admission or at least two outpatient encounters due to any of comorbidities before index date were identified as suffering the comorbidity.

**Figure 1:**
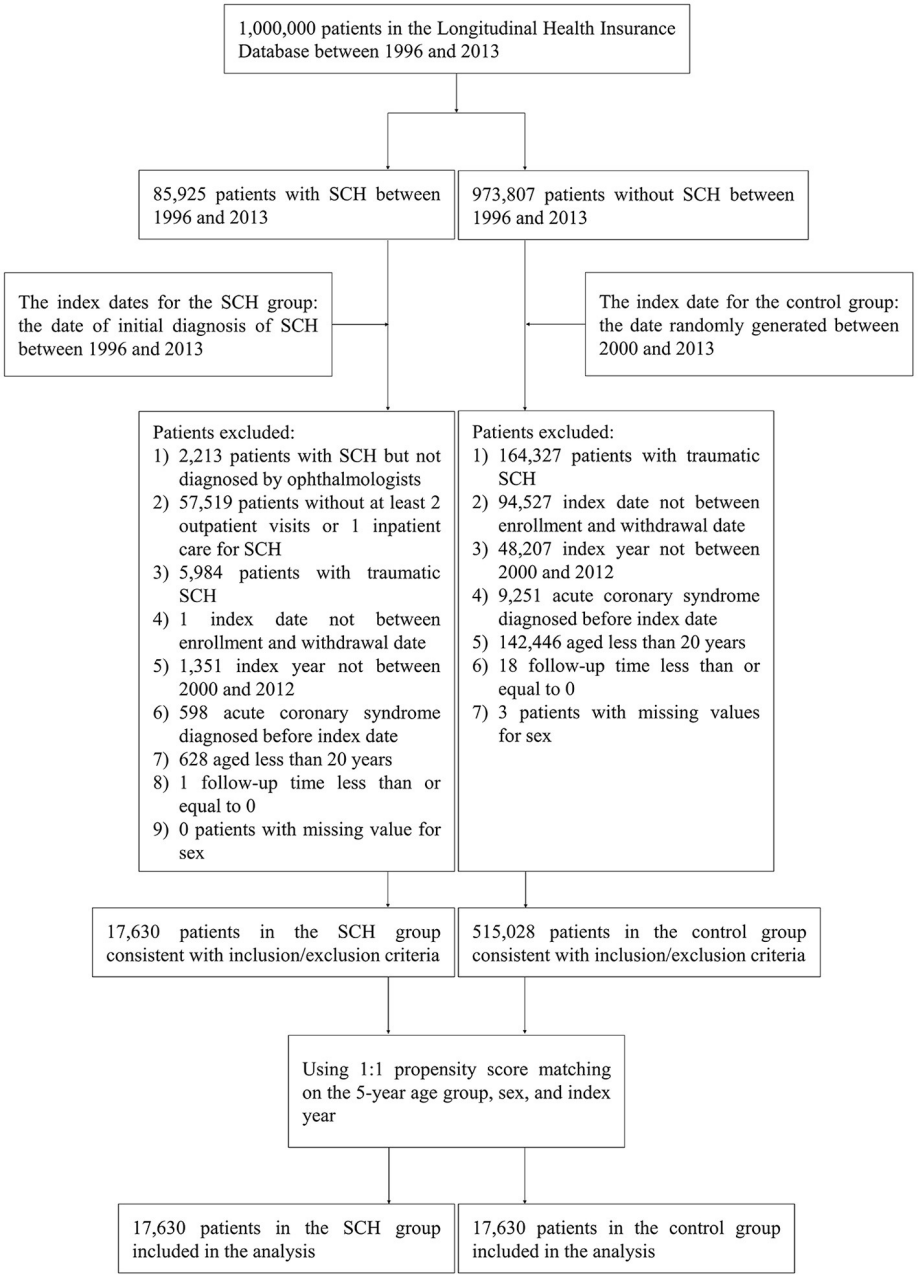
Flowchart of the study population selection.

Controls were randomly matched to cases in ratio of 1:1 using propensity score matching on 5-year age group, gender and index year. Figure 1 displays the flowchart of the study population selection.

### Statistical Analysis

Categorical variables were summarized by counts and percentages; continuous variables were summarized by means and standard deviations (SDs). Standardized mean differences (SMDs) were performed to test for differences in distributions of variables between cases and controls. Cumulative incidence curves were estimated by the Kaplan-Meier method, and the difference between curves was examined by the log-rank test. The incidence rate was calculated as the number of events divided by the person-time-at-risk throughout the follow-up period. Hazard ratios (HRs) with 95% confidence intervals (95% CIs) were computed from univariate Cox proportional hazards models, and adjusted hazard ratios (aHRs) with 95% CIs were computed from multivariate Cox proportional hazards models with covariates of age, gender, comorbidities, and medication. The significant level was set at 0.05. Statistical analyses were accomplished by the SAS 9.4 software (SAS Institute Inc., Cary, NC).

## Results

**Figure 2:**
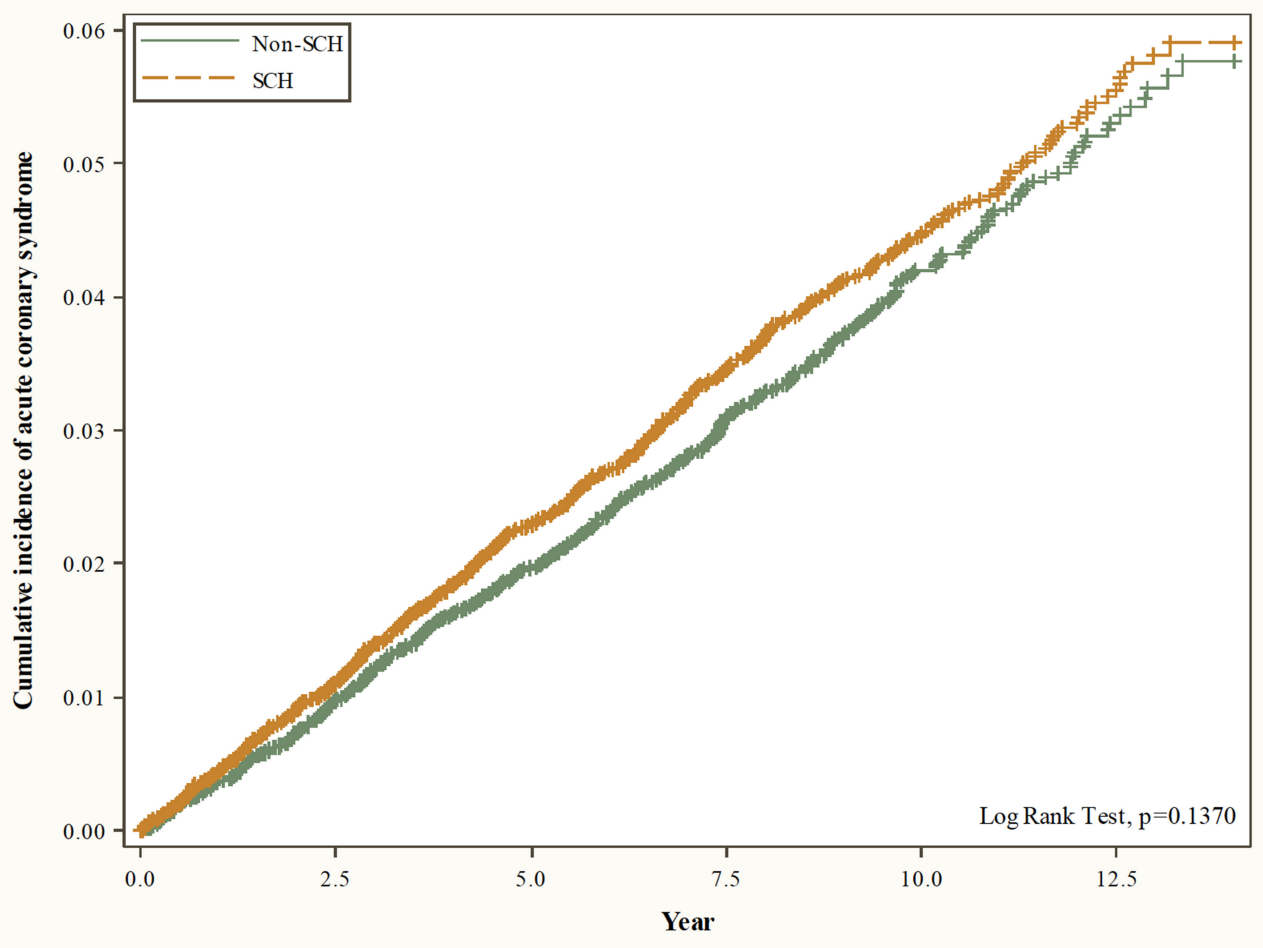
Cumulative incidence of acute coronary syndrome in patients with and without SCH using the Kaplan-Meier method.

Table 1 shows the characteristics of the patients with and without SCH. SCH was more common in the 40–59 age group (53.82%) and females (58.66%). There were notable differences between the two groups for the incidence of COPD (21.39 vs. 25.63%, SMD = 0.1000) and chronic liver disease (14.27 vs. 18.76%, SMD = 0.1214) comorbidities. The average follow-up time was 7.5 ± 3.7 years for the controls and 7.7 ± 3.6 years for the cases. Figure 2 shows the cumulative incidence of ACS in patients with and without SCH using the Kaplan-Meier method. It reveals that there was no significant difference in the cumulative incidence of ACS between the controls and the cases (*p* = 0.1370).

**Table 1:** Characteristics of patients with and without SCH.

**Variable**	**Total**	**Non-SCH**	**SCH**	**SMD^§^**
	***N* = 35,260**	***N* = 17,630**	***N* = 17,630**	
	***n***	***n* (%)/Mean ± SD**	***n* (%)/Mean ± SD**	
Age (year)				
20–39	5,276	2,638 (14.96)	2,638 (14.96)	0.0000
40–59	18,978	9,489 (53.82)	9,489 (53.82)	0.0000
≥60	11,006	5,503 (31.21)	5,503 (31.21)	0.0000
Mean ± SD		53.5 ± 13.4	53.6 ± 13.3	0.0058
Sex				
Female	20,682	10,341 (58.66)	10,341 (58.66)	0.0000
Male	14,578	7,289 (41.34)	7,289 (41.34)	0.0000
Baseline comorbidities				
COPD	8,289	3,771 (21.39)	4,518 (25.63)	0.1000
Diabetes mellitus	4,943	2,387 (13.54)	2,556 (14.50)	0.0276
Hypertension	10,873	5,196 (29.47)	5,677 (32.20)	0.0591
Hyperlipidemia	1,511	673 (3.82)	838 (4.75)	0.0462
Chronic kidney disease	1,084	470 (2.67)	614 (3.48)	0.0473
Chronic liver disease	5,823	2,515 (14.27)	3,308 (18.76)	0.1214
Hemorrhagic condition	257	84 (0.48)	173 (0.98)	0.0594
Baseline medication				
Aspirin	7,694	3,658 (20.75)	4,036 (22.89)	0.0519
Follow-up duration (year)		7.5 ± 3.7	7.7 ± 3.6	0.0550

§ *A standardized mean difference of ≤ 0.1 indicates a negligible difference between the two cohorts*.

*COPD, Chronic Obstructive Pulmonary Disease; SCH, SubConjunctival Hemorrhage; SD, Standard Deviation; SMD, Standardized Mean Difference*.

Table 2 shows the Cox regression analyses of risk factors associated with ACS among all patients. There was no significant difference in the risk of ACS between non-SCH and SCH patients (aHR = 1.02, 95% CI = [0.91, 1.14]). ACS was increasingly common with advancing age (40–59 age group: aHR = 4.39, 95% CI = [2.86, 6.76]; ≥60 age group: aHR = 9.59, 95% CI = [6.21, 14.81]). Males were more likely to develop ACS compared with females (aHR = 1.57, 95% CI = [1.40, 1.76]). Patients with COPD (aHR = 1.30, 95% CI = [1.15, 1.48]), diabetes mellitus (aHR = 1.58, 95% CI = [1.38, 1.81]), hypertension (aHR = 1.71, 95% CI = [1.49, 1.96]), hyperlipidemia (aHR = 1.27, 95% CI = [1.02, 1.58]), or chronic kidney disease (aHR = 1.44, 95% CI = [1.16, 1.79]) were at higher risk of developing ACS. However, patients with hemorrhagic disease were at lower risk of ACS (aHR = 0.27, 95% CI = [0.09, 0.85]). Patients taking aspirin had a higher risk of developing ACS (aHR = 1.67, 95% CI = [1.47, 1.90]).

**Table 2:** Cox regression analyses of risk factors associated with acute coronary syndrome among patients.

**Variable**	**Event**	**Person-year**	**IR**	**Crude**		Adjusted^*^	
	***n* = 1,177**		**1,000 person-years**	**HR (95% CI)**	***p***	**HR (95% CI)**	***p***
**SCH**							
No	555	132,018	4.20	1 (Reference)		1 (Reference)	
Yes	622	135,575	4.59	1.09 (0.97, 1.22)	0.1376	1.02 (0.91, 1.14)	0.7630
**Age (year)**							
20–39	22	43,363	0.51	1 (Reference)		1 (Reference)	
40–59	425	149,644	2.84	5.61 (3.66, 8.61)	<0.0001	4.39 (2.86, 6.76)	<0.0001
≥60	730	74,586	9.79	19.49 (12.76, 29.79)	<0.0001	9.59 (6.21, 14.81)	<0.0001
**Sex**							
Female	563	158,801	3.55	1 (Reference)		1 (Reference)	
Male	614	108,793	5.64	1.59 (1.42, 1.79)	<0.0001	1.57 (1.40, 1.76)	<0.0001
**Baseline comorbidities**							
COPD							
No	737	212,580	3.47	1 (Reference)		1 (Reference)	
Yes	440	55,014	8.00	2.33 (2.07, 2.62)	<0.0001	1.30 (1.15, 1.48)	<0.0001
Diabetes mellitus							
No	822	235,648	3.49	1 (Reference)		1 (Reference)	
Yes	355	31,946	11.11	3.22 (2.84, 3.64)	<0.0001	1.58 (1.38, 1.81)	<0.0001
Hypertension							
No	479	195,241	2.45	1 (Reference)		1 (Reference)	
Yes	698	72,353	9.65	3.98 (3.54, 4.48)	<0.0001	1.71 (1.49, 1.96)	<0.0001
Hyperlipidemia							
No	1,084	258,782	4.19	1 (Reference)		1 (Reference)	
Yes	93	8,812	10.55	2.54 (2.06, 3.15)	<0.0001	1.27 (1.02, 1.58)	0.0309
Chronic kidney disease							
No	1,085	261,341	4.15	1 (Reference)		1 (Reference)	
Yes	92	6,253	14.71	3.58 (2.89, 4.43)	<0.0001	1.44 (1.16, 1.79)	0.0011
Hemorrhagic condition							
No	1,174	266,077	4.41	1 (Reference)		1 (Reference)	
Yes	3	1,517	1.98	0.45 (0.15, 1.40)	0.1683	0.27 (0.09, 0.85)	0.0255
**Baseline medication**							
Aspirin							
No	680	218,389	3.11	1 (Reference)		1 (Reference)	
Yes	497	49,205	10.10	3.29 (2.93, 3.70)	<0.0001	1.67 (1.47, 1.90)	<0.0001

*CI, Confidence Interval; COPD, Chronic Obstructive Pulmonary Disease; HR, Hazard Ratio; IR, Incidence Rate; SCH, Sub Conjunctival Hemorrhage*.

* *Adjusted for age, sex, comorbidity of COPD, diabetes mellitus, hypertension, hyperlipidemia, chronic kidney disease and hemorrhagic condition, and medication of aspirin*.

Table 3 compares the incidence of ACS between patients with and without SCH in different stratifications. It was found that there were no significant differences for any stratifications between non-SCH and SCH patients.

**Table 3:** Comparison of incidence of acute coronary syndrome between patients with and without SCH in different stratifications.

**Variable**	**Non-SCH**			**SCH**			**SCH: Non-SCH**			
	**Event**	**Person-year**	**IR**	**Event**	**Person-year**	**IR**	**Crude**		Adjusted^*^	
	***n* = 561**		**1,000 person-years**	***n* = 619**		**1,000 person-years**	**HR (95% CI)**	***p***	**HR (95% CI)**	***p***
**All**	555	132,018	4.20	622	135,575	4.59	1.09 (0.97, 1.22)	0.1376	1.02 (0.91, 1.14)	0.7630
**Age (year)**										
20–39	7	21,502	0.33	15	21,861	0.69	2.11 (0.86, 5.17)	0.1034	1.93 (0.78, 4.75)	0.1527
40–59	200	74,218	2.69	225	75,427	2.98	1.11 (0.91, 1.34)	0.3038	1.02 (0.84, 1.23)	0.8415
≥60	348	36,299	9.59	382	38,288	9.98	1.04 (0.90, 1.20)	0.6055	0.99 (0.86, 1.15)	0.9319
**Sex**										
Female	261	78,725	3.32	302	80,076	3.77	1.14 (0.96, 1.34)	0.1277	1.08 (0.92, 1.28)	0.3402
Male	294	53,293	5.52	320	55,499	5.77	1.04 (0.89, 1.22)	0.6021	0.96 (0.82, 1.13)	0.6231
**Baseline comorbidities**										
COPD										
No	347	107,623	3.22	390	104,957	3.72	1.15 (1.00, 1.33)	0.0568	1.12 (0.97, 1.30)	0.1139
Yes	208	24,395	8.53	232	30,619	7.58	0.89 (0.73, 1.07)	0.2057	0.87 (0.72, 1.05)	0.1340
Diabetes mellitus										
No	390	116,973	3.33	432	118,675	3.64	1.09 (0.95, 1.25)	0.2143	1.02 (0.89, 1.17)	0.8194
Yes	165	15,046	10.97	190	16,900	11.24	1.02 (0.83, 1.26)	0.8304	1.01 (0.82, 1.24)	0.9423
Hypertension										
No	241	98,345	2.45	238	96,896	2.46	1.00 (0.83, 1.19)	0.9882	0.95 (0.79, 1.14)	0.5835
Yes	314	33,673	9.32	384	38,680	9.93	1.06 (0.92, 1.24)	0.4090	1.06 (0.91, 1.23)	0.4485
Hyperlipidemia										
No	516	128,231	4.02	568	130,551	4.35	1.08 (0.96, 1.22)	0.2052	1.01 (0.90, 1.14)	0.8784
Yes	39	3,787	10.30	54	5,024	10.75	1.06 (0.70, 1.59)	0.7977	1.07 (0.70, 1.61)	0.7627
Chronic kidney disease										
No	518	129,311	4.01	567	132,029	4.29	1.07 (0.95, 1.21)	0.2594	1.00 (0.89, 1.13)	0.9872
Yes	37	2,707	13.67	55	3,546	15.51	1.14 (0.75, 1.72)	0.5490	1.23 (0.80, 1.87)	0.3416
Hemorrhagic condition										
No	554	131,552	4.21	620	134,525	4.61	1.09 (0.98, 1.23)	0.1258	1.02 (0.91, 1.14)	0.7560
Yes	1	467	2.14	2	1,050	1.90	0.92 (0.08, 10.13)	0.9444	0.57 (0.03, 9.84)	0.6986
**Baseline medication**										
Aspirin										
No	320	109,382	2.93	360	109,007	3.30	1.13 (0.97, 1.31)	0.1235	1.06 (0.91, 1.23)	0.4554
Yes	235	22,637	10.38	262	26,569	9.86	0.95 (0.80, 1.13)	0.5765	0.96 (0.80, 1.14)	0.6146

* *Adjusted for age, sex, comorbidity of COPD, diabetes mellitus, hypertension, hyperlipidemia, chronic kidney disease and hemorrhagic condition, and medication of aspirin*.

*CI, Confidence Interval; COPD, Chronic Obstructive Pulmonary Disease; HR, Hazard Ratio; IR, Incidence Rate; SCH, SubConjunctival Hemorrhage*.

## Discussion

To the best of our knowledge, this was the first nationwide, population-based study to evaluate the association between SCH and ACS. Although many patients and clinicians have suspected that SCH could be an early sign of ACS, our adjusted data revealed that there was no significant association between these two diseases.

A previous study with 8,726 subjects by Fukuyama et al. (9) reported that the incidence rate of SCH was 2.9% (*n* = 225), and no significant sexual or age difference was found. However, this study still first indicated the risk factors of SCH, such as systemic hypertension, diabetes. Our data, which is more recent, and other studies (10, 11) have shown that the incidence of both ACS and SCH increase with age, ACS especially (12). It is generally believed that systemic hypertension is a significant cause of SCH in the elderly, while contact-lens-induced injury is the primary cause of SCH in younger patients (13). Another article by Mimura et al. (11), which studied a total of 161 SCH patients, whose age varied from 1 to 94 years old, showed that the peak age of SCH onset was between 61 and 70 years. The elastic fibers and other connective tissues under the conjunctiva tend to become more fragile with age (1), which also supports the theory that SCH is more predominant in the elderly and explains why it has an age-related increase. As for ACS, analyses of data from the United Kingdom revealed that more than twice as many individuals >75 years of age (*n* = 55,028) died from ischemic heart disease, a kind of ACS, compared with younger individuals <75 years (*n* = 25,540) (14).

According to previous research, SCH (15) can be further divided into traumatic and spontaneous SCH, depending on its cause (16). Traumatic SCH may be attributed to the wearing of contact lens or other eye injuries, while spontaneous SCH is more common in older patients. This was observed in our data. Furthermore, previous research has shown that female ethnic groups are more vulnerable to ACS compared with males (17). Our data also showed that the HRs were higher in males, and that females had more SCH cases than males, with the incidence rate increasing with age.

Clinical risk factors for the disease are always a focus of research. A meta-analysis by Dong et al. (10) reported that diabetes is a risk factor for ACS in both women and men. A case-controlled study by Linares et al. (15) found that the OR of ACS was notably higher in those patients with hypertension, diabetes mellitus or a history of smoking. The risk factors for SCH include arteriosclerosis, hyperlipidemia, diabetes, and systemic hypertension (1, 4), and a study by Mimura et al. (13) reported that the primary risk factor was hypertension. Furthermore, a study by Pitts et al. (18) demonstrated that spontaneous SCH is a sign of hypertension.

As you can see, there are a few common risk factors between ACS and SCH. According to our data, risk factors such as diabetes, mellitus, hypertension, and hyperlipidemia have a significant association with ACS. It should be noted that Hubacek et al. reported that total cholesterol might not be a risk factor for ACS (19). In addition, cardiovascular disease is also regarded as a risk factor for SCH. For this reason, many patients suspect that their SCH is an early sign of ACS. However, we believe that one of the main reasons why cardiovascular disease has a significant relationship with SCH is because the common treatments for coronary heart disease are aspirin and warfarin, which can cause bleeding (1, 20–22). In addition, people who take these drugs will also pay more attention to their bleeding, and this may increase the number of diagnoses (23).

A meta-analysis by Carmine Pizzi et al. (24) reported that the incidence and mortality rates of ACS are still high, so a proper method for detecting this condition is important. However, after several adjustments, we found that there was no significant relationship between SCH and ACS. Therefore, SCH should not be considered an early sign of ACS.

The use of aspirin has always been widely discussed within the context of cardiovascular disease as it is regarded as a first-line drug for many cardiovascular diseases. A study by Mora et al. (25) indicated that unless contraindicated, aspirin should be used consistently for the secondary prevention of atherosclerotic cardiovascular disease. However, a meta-regression analysis by Nudy et al. (12) suggested that the efficacy of aspirin for the primary prevention of atherosclerotic cardiovascular disease is limited. Besides, it also mentioned that blood pressure control is a more efficient method of treatment. Based on our data, there is a significant association between hypertension and ACS. In addition, according to our data, people who take aspirin have a HR of 1.67 for ACS. Of course, aspirin is widely taken by individuals with cardiovascular related diseases, so there may be a number of interfering factors, which make this a difficult conclusion which needs more detailed discussion/investigation. Furthermore, a study by Thobani et al. (16) reported that the use of aspirin should be a deliberated decision which is taken individually for each patient.

The current study had several limitations. First of all, SCH is divided into two subtypes, however, due to them having the same ICD codes, it was not possible for us to distinguish between them when conducting the statistics. However, as mentioned above, spontaneous SCH mainly occurs in the elderly, and the elderly made up the majority of our data set, so we suppose that this would have only caused minor interference. Second, because of the patient anonymity policy within the NHIRD, we could not confirm the patients' diagnoses. However, the main diagnostic codes of SCH, ACS, hypertension etc. have been validated within the NHIRD, and have been revealed as highly accurate, so this should not be a major concern. Third, our data was lacking granular data on clinical characteristics, such as smoking status. However, our data did specify whether the patient had COPD, a disease that has been confirmed as having a significant association with smoking. We believe that this could reduce the interference of this limitation. Finally, our study only enrolled Taiwanese people, so it is unclear whether the results can be extrapolated to other ethnicities or populations. Further investigation is needed to determine this.

Despite several limitations, we believe there were some valuable advantages to the current study. First, this was the first study to investigate the potential association between SCH and ACS, which seemed to be highly probable from a clinical perspective. However, after a full evaluation, we determined that these two diseases do not have a significant association with each other. Second, it was a nationwide, population-based study which used the entire 23,000,000 population of Taiwan as the mother group, which is also known as the NHIRD (26–32), and thus minimizes selection bias.

In conclusions, the results of this 14 years cohort study of 35,260 individuals, found that there were no significant differences for any stratifications between non-SCH and SCH patients. In other words, when encountering patients with SCH in the future, doctors should not do too much inspection to avoid medical unnecessary and cause patient anxiety.

## Data Availability Statement

The original contributions presented in the study are included in the article/supplementary material, further inquiries can be directed to the corresponding authors.

## Ethics Statement

The studies involving human participants were reviewed and approved by China Medical University and Hospital (CMUH104-REC2-115(AR-4)). Written informed consent for participation was not required for this study in accordance with the national legislation and the institutional requirements.

## Author Contributions

M-YH, J-NL, K-CH, and JW are responsible for critical comment and revision. P-HC, Y-CC, and K-CH are responsible for manuscript preparation. All authors responsible for research preparation.

## Funding

This study was supported in part by the Ministry of Science and Technology of Taiwan (Grant No. 109-2636-E-040-001, Columbus program of MOST Young Scholar Fellowship). Besides, the authors would like to thank Management Office for Health Data, China Medical University Hospital for their providing of the data.

## Conflict of Interest

The authors declare that the research was conducted in the absence of any commercial or financial relationships that could be construed as a potential conflict of interest.

## Publisher's Note

All claims expressed in this article are solely those of the authors and do not necessarily represent those of their affiliated organizations, or those of the publisher, the editors and the reviewers. Any product that may be evaluated in this article, or claim that may be made by its manufacturer, is not guaranteed or endorsed by the publisher.
